# Impact of Foot Reflexology Versus Simple Massage on Vital Signs, Anxiety, and Pain During Blood Transfusion Among Children With Thalassemia

**DOI:** 10.7759/cureus.67876

**Published:** 2024-08-26

**Authors:** Sunita K Jadhav, Prakash M Naregal, Vaishali R Mohite, Rajashri B Karale

**Affiliations:** 1 Child Health Nursing, Krishna Institute of Nursing Sciences, Krishna Vishwa Vidyapeeth (Deemed to be University), Karad, IND; 2 Medical Surgical Nursing, Krishna Institute of Nursing Sciences, Krishna Vishwa Vidyapeeth (Deemed to be University), Karad, IND

**Keywords:** foot reflexology, blood transfusion, pain, anxiety, vital signs, thalassemia, simple massage

## Abstract

Introduction

Children with thalassemia often require repeated invasive treatments and frequent hospitalizations, resulting in pain, anxiety, and altered vital signs. Implementing non-invasive, non-pharmacological, and inexpensive complementary practices can benefit both the child and their family.

Aim

This study aimed to evaluate the impact of foot reflexology versus simple massage on vital signs, anxiety, and pain induced by blood transfusions in children with thalassemia.

Materials and methods

An experimental study was conducted on 60 children with thalassemia; children aged 2-13 years were selected by systematic random sampling. The participants were separated into two groups: 30 children received foot reflexology and 30 children received a simple massage. Data were collected using a self-structured demographic profile, vital signs record sheet, standard Observational Scale of Behavioral Distress-Revised (OSBD-R) scale, and visual analog scale (VAS). Paired and unpaired t-tests were used to evaluate the effects of the interventions. The chi-square test was utilized to evaluate the relationship between demographic and dependent variables.

Result

Foot reflexology showed a significant difference (P < 0.05) in systolic and diastolic blood pressure and a highly significant difference (P < 0.0001) in anxiety and pain. The simple massage group showed a significant effect on temperature, anxiety, and pain. Both groups demonstrated a significant impact (P < 0.05) on systolic blood pressure and pain after the intervention.

Conclusion

Most children were diagnosed with thalassemia during infancy, had a history of both parent’s thalassemia minor, and were Rh+ve. Foot reflexology was more effective in reducing anxiety and pain than simple massage. Additionally, foot reflexology had a significant effect on systolic and diastolic blood pressure, while simple massage significantly affected temperature in children with thalassemia.

## Introduction

Thalassemia is a genetic blood disorder affecting approximately 15 million people worldwide, with around 240 million individuals carrying the thalassemia trait. Every year, approximately 100,000 children worldwide are born with thalassemia major, with India contributing to about 10,000 of these cases [1]. According to the National Health Mission (2016) report, India is estimated to have between 1,00,000 and 1,50,000 children diagnosed with thalassemia major, while nearly 42 million individuals worldwide are carriers of the β thalassemia trait [1]. Thalassemia major, or Cooley’s anemia, occurs when a person inherits a variant gene from both parents. Although infants with this condition are typically healthy at birth, symptoms emerge around six months of age. The disease is characterized by moderate to severe anemia, requiring frequent blood transfusions and continuous medical care. It can significantly impact lifespan and overall quality of life. Thalassemia minor, or beta thalassemia trait, occurs when a person inherits a variant gene from one parent. Carriers typically have no symptoms or only mild anemia and usually do not require treatment.

According to the Thalassemia Foundation of Canada, the genetic defect in thalassemia results in severe anemia and bone marrow expansion as the body tries to produce more red blood cells. Low hemoglobin levels reduce blood’s ability to carry oxygen, causing fatigue and shortness of breath and impacting overall oxygen levels. Regular blood transfusions and iron chelation therapy are essential to prevent severe anemia. Transfusions raise hemoglobin levels, reducing anemia [2]. Thalassemia is a hereditary blood disorder caused by mutations in the genes that produce hemoglobin, the oxygen-carrying protein in red blood cells. These genetic changes lead to reduced or defective hemoglobin production, resulting in the breakdown of red blood cells and causing severe anemia. Children diagnosed with beta-thalassemia major frequently need to undergo repeated invasive procedures, like venipuncture or blood transfusions, and frequent hospitalizations [3]. During hospitalization, children often experience separation anxiety from family, activity restrictions, pain from procedures, side effects of iron chelation therapy, and fear of mortality. These factors can contribute to significant anxiety and distress in children [4]. Iron chelation therapy eliminates excess iron from the body and is commonly used for patients with conditions like thalassemia who undergo frequent blood transfusions. Common chelating agents include deferoxamine, deferasirox, and deferiprone.

In the past, foot reflexology and other alternative therapies were historically perceived as traditional, non-scientific remedies predominantly embraced by individuals with lower levels of education, belonging to lower socioeconomic classes, and among the elderly. However, as foot reflexology gains popularity among patients and healthcare professionals alike, there is a growing effort to scientifically evaluate its effectiveness [5]. Foot reflexology uses pressure on specific spots on the feet that are thought to connect to different body parts, aiming to improve overall health. Simple massage, on the other hand, involves general, gentle rubbing of muscles to help with relaxation without focusing on specific areas. Reflexology operates on the principle that the soles of the feet mirror the entire body. Through thumb and finger pressure on specific reflex points, each corresponding to different body parts, reflexologists aim to promote overall well-being. These reflex points range from the distal phalanx of the big toe (representing the head) to the calcaneum (representing the pelvis) [6]. The use of non-invasive, non-pharmacological, simple, and cost-effective nursing practices provides substantial benefits for children and their families, alleviating the financial strain on parents [7]. Many studies highlight the impact of foot reflexology on pain reduction. Integrating massage therapy into nursing care enhances holistic patient management. Emphasizing these complementary therapies, which nurses can administer independently, supports nursing practice autonomy and patient care quality [6]. This topic has not been studied in India before. Therefore, the researcher felt the need to undertake the current study to contribute to the body of knowledge in nursing and to help children suffering from thalassemia.

The following are the objectives of this study: (1) to assess the vital signs, level of anxiety, and level of pain of children with thalassemia receiving blood transfusion; (2) to determine the effect of foot reflexology versus simple massage on vital signs, anxiety, and pain produced by blood transfusion in children with thalassemia; and (3) to find the association between vital signs, level of anxiety, and level of pain in children receiving blood transfusion with selected demographic variables.

## Materials and methods

A quantitative research approach and experimental research design were used to study the effect of foot reflexology and simple massage on vital signs, anxiety, and pain produced by blood transfusion in children with thalassemia admitted to a pediatric ward in selected tertiary care hospitals in western Maharashtra, India. Research tools include socio-demographic variables, such as age, gender, residence, religion, socio-economic status, age of diagnosis (in years and months), family history of thalassemia, blood transfusion starting age, blood group, Rhesus factor, total number of blood transfusion cycles received, vital signs record sheet to check vital signs, digital thermometer, watch, ISOMED stethoscope, sphygmomanometer, and pulse oximeter. An Observational Scale of Behavioral Distress-Revised (OSBD-R) evaluates children experiencing anxiety when receiving medical care, using eight behaviors: crying, screaming, physical and verbal resistance, seeking parental help, requesting information, predicting pain, and flailing. Each behavior is scored 0-4 (0.5 for observed; 0 for absent). Scores range from <1 (no anxiety), 1.5-2 (mild), 2.5-3 (moderate), to 3.5-4 (severe), and Elliott et al. validated its reliability and validity [8]. The visual analog scale (VAS) for pain uses a horizontal line with emoji indicators to measure pain from 0 to 10. Scores are interpreted as 1-4 (mild pain), 5-6 (moderate pain), and 7-10 (severe pain), and Begum and Hossain validated its reliability and validity [9].

Sample size

The sample size was calculated based on a previous research study conducted by Dehghanmehr et al. [10].

n = (SD^2^_C_ + SD^2^_E_) × 7.8/(M_C_ − M_E_)^2^

= (1.40^2^ + 0.63^2^) × 7.84/(2.18 − 0.70)^2^

= (1.96 + 0.39) × 7.84/(1.48)^2^

= 2.35 x 7.84/2.19

= 18.42/2.19

= 8.4

So, the minimum sample size is 9.

Two groups of 60 kids were created using a systematic random sampling technique. For this study, three hospitals were selected using the lottery method. From each hospital, 20 children with thalassemia were chosen. Of these, 10 children were assigned to the foot reflexology group and 10 to the simple massage group, using systematic random sampling. According to the inclusion criteria, the first child was placed in the foot reflexology group and the second in the simple massage group. The foot reflexology group consists of 30 children, and the simple massage group consists of 30 children. Inclusion criteria include children with thalassemia aged 2-13 years who have come for blood transfusion. Exclusion criteria include children in critical condition who have complications other than thalassemia, undergoing anti-anxiety drug treatment, swelling, and unhealed wounds or cuts over their feet.

Data collection

After obtaining ethical clearance, prior authorization was acquired from the relevant authorities of Krishna Hospital and Medical Research Center, Karad, India; Padma Bhushan Vasantdada Patil Government Hospital, Sangli, India; and Government Medical College and Hospital, Miraj, India. The sampling was done using a systematic random sampling technique. The researcher introduced herself, established good rapport, explained the purpose of the study, received parents’ informed consent, and collected socioeconomic data of all participants. The researcher used OSBD-R to observe children’s behavior and the VAS for facial expressions (pain). Vital indicators, such as body temperature, heart rate, breathing rate, blood pressure (systolic and diastolic), and SPO2 levels, were recorded before interventions using a vital signs record sheet. Foot reflexology group children were positioned comfortably. The researcher washed hands and then administered a 20-minute foot reflexology (10 minutes per foot), focusing on the relaxation of specific points: foot, solar plexus, pituitary, heart, liver, and great surge point. In a simple massage group, the legs were warmed up. Using the palm and fingers of one hand, the ankle, sole, back of the foot, and toes were massaged for 20 minutes. Blood transfusion started, and the child was reassessed using OSBD-R, VAS, and vital signs post-transfusion. Data analysis involves data being compiled into a master sheet. Frequency and percentage were used to describe the demographic factors. Both paired and unpaired t-tests were used to compare mean scores. The chi-square test was used to evaluate the relationship between dependent variables and sociodemographic characteristics. Results were visually presented through tables and graphs. Confidentiality of the data about children and their parents was maintained throughout the informed consent process and interview.

## Results

Frequency and distribution of demographic variables

Table 1 shows that the majority (46.7%) of children undergoing foot reflexology were aged 2-5 years, predominantly male (73.3%), from rural areas (66.7%), and Hindu (90%). About 36.7% were from lower-middle socioeconomic backgrounds. Most were diagnosed by the age of 3 (93.3%), with 56.7% diagnosed in infancy. In all, 46% had parents with thalassemia minor. Rhesus factor was Rh+ve in 96%, and 40% had blood group O+ve.

**Table 1:** Frequency and distribution of demographic variables F, frequency; BT, blood transfusion; RH, Rhesus

Demographic characteristics	Foot reflexology	Simple massage
	F (%)	F (%)
Age in years		
2-5	14 (46.7)	11 (36.7)
6-9	8 (26.7)	8 (26.7)
10-13	8 (26.7)	11 (36.7)
Gender		
Male	22 (73.3)	19 (63.3)
Female	8 (26.7)	11 (36.7)
Residence		
Urban	10 (33.3)	8 (26.7)
Rural	20 (66.7)	22 (73.3)
Religion		
Hindu	27 (90)	25 (83.3)
Muslim	3 (10)	5 (16.7)
Socioeconomic status		
Upper	2 (6.7)	0
Upper-middle	9 (30)	8 (26.7)
Lower-middle	11 (36.7)	18 (60)
Lower	8 (26.7)	4 (13.3)
Age of diagnosis in years		
0-3	28 (93.3)	26 (86.7)
4-7	1 (3.3)	4 (13.3)
8 and above	1 (3.3)	0
Age of diagnosis in months		
0-12	17 (56.7)	16 (53.3)
13-24	9 (30)	7 (23.3)
25-36	2 (6.7)	3 (10)
37 and above	2 (6.7)	4 (13.3)
Family history		
Both parent’s thalassemia minor	14 (46.7)	9 (30)
Siblings with thalassemia	0	3 (10)
Both parents and siblings with thalassemia	4 (13.3)	6 (20.0)
Consanguineous marriage	3 (10)	7 (23.3)
Not tested	5 (16.7)	2 (6.7)
Both parents’ thalassemia minor and consanguineous marriage	4 (13)	3 (10)
Age of starting BT		
0-5 years	28 (93.3)	30 (100)
6-10 years	2 (6.7)	0
Blood group		
A +ve	7 (23.3)	9 (30)
B +ve	9 (30)	9 (30)
AB +ve	1 (3.3)	1 (3.3)
O +ve	12 (40)	9 (30)
O −ve	1 (3.3)	2 (6.7)
RH factor		
Positive +ve	29 (96.66)	28 (93.33)
Negative −ve	1 (3.3)	2 (6.7)
BT cycles		
1-5	4 (13.3)	1 (3.3)
6-10	0	1 (3.3)
11-20	9 (30)	6 (20.0)
21 and more	17 (56.7)	22 (73.3)

In the simple massage group, the majority of children (36.7%) fell within the 2-5 years and 10-13 years age brackets, with a notable male predominance (63.3%) and a significant rural residence (73.3%). Most identified with the Hindu religion (83.3%) and from lower-middle socioeconomic backgrounds (60%). Diagnosis occurred predominantly by the age of 3 (86.7%) and infancy (53.3%), with a high prevalence (63.33%) of parental thalassemia minor or consanguineous marriage. The vast majority were Rh+ve (93.33%), with blood groups O+ve, A+ve, and B+ve, each comprising 30% of the group.

Comparison of mean scores of dependent variables in the foot reflexology group

Table 2 shows the significant impact of foot reflexology on systolic and diastolic blood pressure (P < 0.05) and highly significant effects on anxiety and pain levels (P = 0.001). Anxiety levels increased from 4.03 (±0.80) to 3.2 (±0.84), and the level of pain significantly decreased from 2.46 (±0.57) to 1.76 (±0.67) after foot reflexology.

**Table 2:** Comparison of the impact of foot reflexology on dependent variables NS, not significant; S, significant; HS, highly significant; SD, standard deviation; BP, blood pressure

Variable	Before foot reflexology (Mean ± SD)	After foot reflexology (mean ± SD)	P-value	Paired t-test	Result
Temperature	98.33 ± 0.24	98.3 ± 0.21	0.70306	0.384	NS
Pulse rate	109.4 ± 12.56	105.0 ± 14.08	2.49	5.836	NS
Respiratory rate	23.13 ± 2.08	22.87 ± 1.94	0.25	1.161	NS
Systolic BP	105.3 ± 9.37	102.67 ± 8.68	0.01	2.504	S
Diastolic BP	69.6 ± 4.90	67.00 ± 5.35	0.01	2.504	S
SpO2	98 ± 0.58	98.20 ± 0.61	0.136	1.533	NS
Anxiety	4.03 ± 0.80	3.2 ± 0.84	0.0001	9.89	HS
Pain	2.46 ± 0.57	1.76 ± 0.67	0.0001	8.226	HS

Comparison of mean scores of dependent variables in simple massage group

Table 3 shows that simple massage has a significant effect on temperature, anxiety, and pain (P < 0.05). The level of anxiety significantly decreased from 3.66 (±0.92) to 3.43 (±0.89), and the level of pain also significantly decreased from 2.26 (±0.63) to 2.1 (±0.66) after simple massage (P = 0.022). The mean before the simple massage was 98.32°F, and after the simple massage, it was 98.4°F, with a P-value of 0.025.

**Table 3:** Comparison of the effect of simple massage on dependent variables NS, not significant; S, significant; HS, highly significant; BP, blood pressure; SD, standard deviation

Variable	Before simple massage (mean ± SD)	After simple massage (mean ± SD)	Paired t-test	P-value	Result
Temperature	98.32 ± 0.21	98.4 ± 0.18	2.346	0.025	S
Pulse rate	105.1 ± 11.95	104.5 ± 11.82	0.941	0.354	NS
Respiratory rate	24.07 ± 8.16	23.60 ± 1.99	0.338	0.737	NS
Systolic BP	107.6 ± 8.97	107.67 ± 9.71	0	1	NS
Diastolic BP	69.3 ± 5.20	69.67 ± 5.56	0.296	0.768	NS
SpO2	97.97 ± 0.61	97.17 ± 0.59	1.533	0.136	NS
Anxiety	3.66 ± 0.92	3.43 ± 0.89	2.971	0.005	S
Pain	2.26 ± 0.63	2.1 ± 0.66	2.408	0.022	S

Comparison of mean scores of foot reflexology and simple massage on vital signs, level of anxiety, and pain

Table 4 reveals that foot reflexology and simple massage have a significant association (P < 0.05) with systolic blood pressure and pain. The level of pain significantly decreased in the foot reflexology group by 1.76 ± 0.67 than in the simple massage group mean by 2.1 ± 0.66 (P = 0.05), and systolic blood pressure significantly decreased in the foot reflexology group by 102.6 mmHg ± 8.6 than in the simple massage group by 107.6 mmHg ± 9.7 (P = 0.040). 

**Table 4:** Comparison of foot reflexology and simple massage on dependent variables NS, not significant; S, significant; HS, highly significant; BP, blood pressure; SD, standard deviation

Variable	After foot reflexology (mean ± SD)	After simple massage (mean ± SD)	Mean difference	T-value	P-value	Result
Temperature	98.3 ± 0.21	98.42 ± 0.18	0.12	1.410	0.164	NS
Pulse rate	105.0 ± 14.0	104.5 ± 11.82	0.5	0.139	0.890	NS
Respiratory rate	22.8 ± 1.94	23.6 ± 1.99	0.8	1.443	0.154	NS
Systolic BP	102.6 ± 8.6	107.6 ± 9.7	5	2.102	0.040	S
Diastolic BP	67.0 ± 5.35	69.6 ± 5.5	2.6	1.893	0.063	NS
SpO2	98.20 ± 0.61	98.17 ± 0.59	0.03	0.215	0.831	NS
Anxiety	3.2 ± 0.84	3.43 ± 0.89	0.23	1.036	0.304	NS
Pain	1.76 ± 0.67	2.1 ± 0.66	0.34	1.926	0.05	S

Association of dependent variables before intervention with selected demographic variables

Vital signs had no association with selected demographic variables. The level of anxiety had a highly significant (P = 0.001) association with age and a significant association with age of diagnosis in months and family history. The level of pain had a highly significant (P = 0.001) association with age and a significant association with age of diagnosis in months (Tables 5, 6).

**Table 5:** Association of the level of anxiety with selected demographic variables HS, highly significant association; SA, significant association; NS, not significant association; F, frequency; BT, blood transfusion

OSBD-R score	Absence of behavior	Lack of anxiety	Mild anxiety	Moderate anxiety	Severe anxiety	Chi-square test	P-value	Result
	F	F	F	F	F			
Age								
2-5 years	0	0	1	7	17	44.65	0.001	HS
9 years	0	0	8	8	0			
10-13 years	0	2	13	4	0			
Gender								
Male	0	2	15	11	13	2.404	0.493	NS
Female	0	0	7	8	4			
Residence								
Urban	0	0	7	5	6	1.241	0.743	NS
Rural	0	2	15	14	11			
Religion								
Hindu	0	2	8	16	16	1.671	0.643	NS
Muslim	0	0	14	3	1			
Socioeconomic status								
Upper class	0	0	0	1	1	5.487	0.79	NS
Upper middle	0	0	8	5	4			
Lower middle	0	2	11	9	7			
Lower	0	0	3	4	5			
Age of diagnosis in years								
0-3 years	0	2	17	18	17	6.741	0.346	NS
4-7 years	0	0	4	1	0			
8 years & above	0	0	1	0	0			
Age of diagnosis in months								
0-12 months	0	1	7	13	12	20.467	0.015	HS
13-24 months	0	1	9	1	5			
25-36 months	0	0	1	4	0			
37 months & more	0	0	5	1	0			
Family history of thalassemia								
Both parents with thalassemia minor	0	1	7	10	5	25.519	0.043	SA
Sibling with thalassemia	0	0	2	0	1			
Both parents & siblings with thalassemia	0	1	4	4	1			
Consanguineous marriage	0	0	1	0	6			
Not tested	0	0	7	1	2			
Both parents with thalassemia minor & consanguineous marriage	0	0	1	4	2			
Blood transfusion starting age								
0-5 years	0	2	21	18	17	0.975	0.807	NS
6-10 years	0	0	1	1	0			
Blood group								
A +ve	0	0	4	7	5	12.14	0.435	NS
B +ve	0	1	9	3	5			
AB +ve	0	0	1	0	1			
O +ve	0	1	8	6	6			
O −ve	0	0	0	3	0			
Blood transfusion cycles								
1-5	0	0	1	3	1	20.9	0.052	SA
6-10	0	0	0	1	0			
11-20	1	0	3	9	2			
<41	0	9	2	9	1			

**Table 6:** Association of the level of pain with selected demographic variables HS, highly significant association; SA, significant association; NS, not significant association; F, frequency

VAS score	Mild pain	Moderate pain	Severe pain	Chi-square test	P-value	Result
	F	F	F			
Age group						
2-5 years	0	2	23	59.074	0	HS
6-9 years	0	13	3			
10-13 years	4	15	0			
Gender						
Male	4	19	18	2.21	0.331	NS
Female	0	11	8			
Residence						
Urban	2	7	9	1.171	0.557	NS
Rural	2	23	17			
Religion						
Hindu	3	26	23	0.544	0.762	NS
Muslim	1	4	3			
Socioeconomic status						
Upper class	0	0	2	4.345	0.63	NS
Upper middle	2	8	7			
Lower middle	2	15	12			
Lower	0	7	5			
Age of diagnosis in years						
0-3 years	3	25	26	5.937	0.204	NS
4-7 years	1	4	0			
8 years & above	0	1	0			
Age of diagnosis in months						
0-12 months	0	14	19	13.446	0.036	NS
13-24 months	3	8	5			
25-36 months	0	3	2			
37 months & more	1	5	0			
Family history						
Both parents with thalassemia minor	2	10	11	9.573	0.479	NS
Sibling with thalassemia	0	2	1			
Both parents & siblings with thalassemia	1	6	3			
Consanguineous marriage	0	1	6			
Not tested	1	7	2			
Both parents with thalassemia minor & consanguineous marriage	0	4	3			
Blood transfusion starting age						
0-5 years	4	28	26	2.069	0.355	NS
6-10 years	0	2	0			
Blood group						
A +ve	1	8	7	3.955	0.861	NS
B +ve	0	10	8			
AB +ve	0	1	1			
O +ve	3	9	9			
O −ve	0	2	1			
Blood transfusion cycles						
1-5	0	1	4	11.662	0.07	NS
6-10	0	0	1			
11-20	0	5	10			
<21	4	24	11			

## Discussion

The study findings revealed that the majority of the children were male, predominantly Hindu, from rural areas, diagnosed during infancy, belonged to the lower-middle class, and had a rhesus factor of Rh+ve. Foot reflexology was shown to have a significant effect on both systolic and diastolic blood pressure and a highly significant effect on reducing anxiety and pain. Simple massage significantly affected temperature, anxiety, and pain. In comparison, after both interventions, foot reflexology had a more significant impact on the reduction of systolic blood pressure and pain than the simple massage.

Simhachalam et al.’s (2018) study on 100 children with thalassemia found that 70% were male, 60% were Hindu, and 45% were from lower-middle-class families [11]. The mean age of diagnosis was 2-19 months. Current study findings support the study conducted by Mansouri et al. (2018) on 60 children with thalassemia under 12 years, showing male predominance and diagnosis by the age of 2 [4]. Systolic and diastolic blood pressure and anxiety scores significantly improved after foot reflexology (P = 0.0001 and 0.003, respectively) and non-specific massage (P = 0.003). The average anxiety level post-intervention was highly significant (P = 0.0001) [4]. A similar finding was reported by Hussein (2022) on blood groups, and rhesus factors were significantly influenced in beta-thalassemia patients undergoing blood transfusions following both interventions. Blood type O was the most common (45%), and there was a significant decrease in Rh−ve samples among thalassemia patients (7%) compared to controls (15%). Rh+ve was 93% in thalassemia patients compared to 85% in controls [12]. The study supports the findings of Ramadan Esmail Magor et al. (2023) that foot reflexology significantly reduces pain and anxiety (P < 0.001) in diabetic children during insulin injections [13]. A similar finding was reported by Ghazavi et al. (2016), showing that foot reflexology significantly reduced systolic and diastolic blood pressure (P = 0.0001) and anxiety (P = 0.0001) in children with leukemia undergoing chemotherapy. In contrast, simple massage did not affect anxiety [14], and a similar finding was reported by Jing et al. (2022). The mean systolic and diastolic blood pressures post-intervention differed significantly between the two groups (P < 0.0001 and p = 0.002, respectively). This indicates that foot reflexology helps reduce blood pressure [15].

Limitations

Children with thalassemia from ages 2-13 years were the only participants in the study. The sample size was limited to 60 children with thalassemia. The current study is limited to the effect of foot reflexology and simple massage on vital signs, level of anxiety, and level of pain.

Strengths and future directions

This study is non-invasive and affordable to lower-income families. For future research, we plan to conduct a study that includes wider age groups so that a spectrum of demographic parameters can be studied. In addition, the study would be planned before the IV cannula insertion among children with thalassemia.

## Conclusions

Implementation of non-pharmacological interventions like foot reflexology and simple massage in the care of children with thalassemia effectively reduces pain and anxiety during blood transfusions. Foot reflexology shows statistically significant benefit, positively affecting systolic blood pressure. Training nurses in these methods is crucial for providing comprehensive and compassionate care that addresses both the physical and emotional needs of these children.
